# 

**Published:** 1916-11-11

**Authors:** 


### CONTENTS.

PAGE

**Clairvoyance and the War** ... ... ... ... ... 109

**The Position of the Territorial Medical Officer** 110

**Hospital and Institutional News** ... ... ... 111

The King and Queen at Sussex Hospitals—Honours for Gardelegen Heroes—Cardiff's Great Day—Sir Edward Nicholl's Ambition—A Ministry of Public Health—A Hospital Romance—The Development of the West London Hospital—The Needs of the Ophthalmic Department—An Improvement Scheme at St. George's—The Details of the Proposals—A Current Fallacy about the Roehampton Hospital—King Manoel's Campaign—The Manchester and Salford Lock Hospital—An Addition to Halifax War Hospital—Should a Hospital Chaplain do Almoner's Work?—A Lincolnshire Tuberculosis Scheme—Public Funds and Doubtful Methods—A New Meaning to "After-Care"—The Germans and Nurse Cavell—A National Institute of Mothercraft—The Edith Cavell Homes of Rest for Nurses—Cutting the Red-Tape Knot—War Charities Accounts—Some Novel Collecting Schemes—The Remuneration of Doctors at Venereal Clinics—Provisions for the Hospitals—The "Rep. Mist." Formula—A Curious Argument Against Closing an Irish Workhouse—A Scottish Hospital Gazette—This Week's Drug Market—To Our Readers.

**The Hospital and the Surgeon** ... ... ... ... 117

II.—Service and Administration.

**England's Recent Progress** .. ... ... ... ... 121

The Refining of Crude Statistics.

PAGE

**The School Doctor** ... ... ... ... ... ... 122

Malnutrition.

**The Institutional Library** ... ... ... ... ... 123

The Nation of the Future—Newsholme's School Hygiene. The Laws of Health in Relation to School Life—A Practical Guide to X-Rays. Electro-Therapeutics and Radium Therapy—Pulmonary Tuberculosis in General Practice—Bed-sores: Their Prevention and Cure—Clinical Notes for Probationers—The Rhymes of a Red Cross Man—Medical Reporting in Pitman's Shorthand—Low's Handbook to the Charities of London, 1916.

**The Matrons' and Sisters' Department** ... 125

II.—Poor-Law Institutions and Nurse-Training.
Round the Hospitals.
Mr. Asquith and Registration—Grave Illness of Miss Haughton—The Nightingale Medal—The Supply of Nursing Assistants—A Weekly Off-Duty Day—Midwives for Canada.

**The Editor's Letter-Box** ... .. ... ... 127

Knighthoods for Hospital Secretaries.

**Gifts and Bequests** ... ... ... ... ... ... 127

**Parliament and the Profession** ... ... ... ... 128

**Matrons' and Sisters' Appointments** ... ... 128

**The Institutional Worker** ... .. (Suppl.) 1

The Supply of Comforts for Wounded and Warriors.
War Conditions and Hospital Expenditure.
Question for November.
Enquiries and Answers.

